# An angiogenesis platform using a cubic artificial eggshell with patterned blood vessels on chicken chorioallantoic membrane

**DOI:** 10.1371/journal.pone.0175595

**Published:** 2017-04-17

**Authors:** Wenjing Huang, Makoto Itayama, Fumihito Arai, Katsuko S. Furukawa, Takashi Ushida, Tomohiro Kawahara

**Affiliations:** 1 Department of Biological Functions Engineering, Kyushu Institute of Technology, Wakamatsu-ku, Kitakyushu, Japan; 2 Department of Micro-Nano Systems Engineering, Nagoya University, Chikusa-ku, Nagoya, Japan; 3 Department of Bioengineering, School of Engineering, The University of Tokyo, Bunkyo-ku, Tokyo, Japan; 4 The Center for Disease Biology and Integrative Medicine, Graduate School of Medicine, The University of Tokyo, Bunkyo-ku, Tokyo, Japan; Universita degli Studi di Bari Aldo Moro, ITALY

## Abstract

The chorioallantoic membrane (CAM) containing tiny blood vessels is an alternative to large animals for studies involving angiogenesis and tissue engineering. However, there is no technique to design the direction of growing blood vessels on the CAM at the microscale level for tissue engineering experiments. Here, a methodology is provided to direct blood vessel formation on the surface of a three-dimensional egg yolk using a cubic artificial eggshell with six functionalized membranes. A structure on the lateral side of the eggshell containing a straight channel and an interlinked chamber was designed, and the direction and formation area of blood vessels with blood flow was artfully defined by channels with widths of 70–2000 μm, without sharply reducing embryo viability. The relationship between the size of interlinked chamber and the induction of blood vessels was investigated to establish a theory of design. Role of negative and positive pressure in the induction of CAM with blood vessels was investigated, and air pressure change in the culture chamber was measured to demonstrate the mechanism for blood vessel induction. Histological evaluation showed that components of CAM including chorionic membrane and blood vessels were induced into the channels. Based on our design theory, blood vessels were induced into arrayed channels, and channel-specific injection and screening were realized, which demonstrated proposed applications. The platform with position- and space-controlled blood vessels is therefore a powerful tool for biomedical research, which may afford exciting applications in studies involved in local stimulation of blood vessel networks and those necessary to establish a living system with blood flow from a beating heart.

## Introduction

The chicken chorioallantoic membrane (CAM) is a highly vascularized extraembryonic membrane that acts as a respiratory system for the development of the chick embryo [1–3]. Since the use of large animals such as pigs and cows in biomedical research is always expensive and raises serious ethical concerns [4–6], limiting their usefulness for experiments, scientists use small animals such as zebrafish, *Xenopus*, and the chick embryo as ethically acceptable alternatives [7–11]. Among the small animal models, the chick embryo and its extraembryonic circulatory system (CAM) is a preferred model, with advantages which can be summarized as follows [11–14]: 1) Using the CAM does not create ethical concerns according to the Animals (Scientific Procedures) Act 1986 [15]. 2) The price of a fertilized chicken egg is much lower than that of a mouse [12, 16]. 3) The CAM is thin and transparent, allowing clear visualization of blood vessel development of blood vessels [17–19], and it shows rapid development of the blood vessel network and slow developmental progression of the immune system [12, 20], which is a benefit for applications related to transplantation [12].

Owing to these advantages, the applications of the CAM include the following: research on vascular development and angiogenesis; on the pathways involved in tumor growth and metastasis; organ transplantation and tissue engineering; a screening platform for distribution and toxicology of anti-cancer drugs; and a platform for the testing of biocompatibility of biomaterials, amongst others [11, 12, 21–24].

The disadvantages of the methods used in the previous studies are as follows: 1) Low observability and accessibility. For the method using natural eggshell with a small hole over the air cell of the egg, there is only a limited CAM area and blood vessels near the body of the chick embryo for observation and tissue culture [25–27]. Only one experimental sample and one result were obtained per egg, and the spreading and development of the front edge of the blood vessel network cannot be observed when the network spreads outside the small window area. Designing the growing axis of the network is difficult, because the eggshell is tough and cannot be seen through, and blood vessels on the CAM are tiny and fragile. For these reasons alone, the tissue inside the eggshell has low accessibility. Therefore, the usage of CAM faces limitations in biomedical research such as drug delivery and screening. 2) Changeable observation area. Previous studies showed that it is not possible to fix cell-matrix constructs onto the surface of the CAM over a definite examination period, and thus the construct could not be continuously studied by light microscopic examination [28]. To solve this problem, Borges et al. designed a cylinder model to fix the position of the cell-ECM construct [28]. However, this is a complex experimental setup and lacks a straightforward approach to quantify the angiogenic response [16]. 3) Randomly distributed blood vessels. A gelatin sponge or tissue implant on the CAM induces only ‘spoked-wheel’ blood vessel formation with random orientation, limiting use of the CAM in research such as organ transplantation and tissue engineering, because organized blood vessels are necessary for the transport of oxygen and nutrients in specific directions and locations [26, 29, 30].

**Fig 1:**
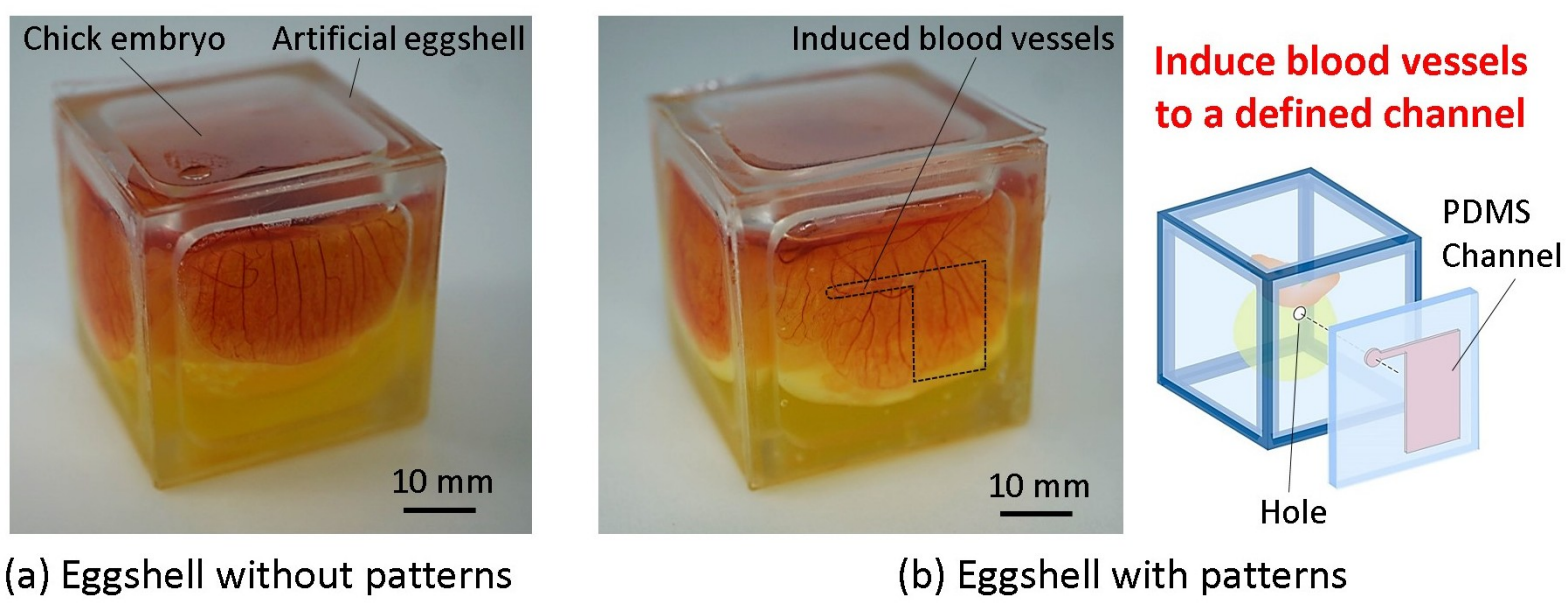
The concept of the current study. (a) A cubic artificial eggshell made of a frame structure and six transparent PDMS membranes was used to culture a chick embryo *in vitro* from embryonic day 3. On day 7, blood vessels on the CAM spread onto the lateral side membrane without specific orientation. (b) The current study established a defined area for inducing and arranging blood vessels with blood flow on the lateral side membrane of the cubic eggshell, for biomedical research such as CAM local stimulation and tissue engineering.

To solve these problems, we proposed a design and a fabrication method for a cubic artificial eggshell in a previous study using an oxygen-permeable membrane surface [31, 32]. The cubic eggshell was fabricated by integrating a polycarbonate (PC) frame structure and six dimethylpolysiloxane (PDMS) membranes. A chick embryo was transferred into the cubic eggshell after a 3-day incubation period. Blood vessels on the CAM were observed not only on the top face but also on the lateral membranes of the cubic eggshells (Fig 1a, S1 Movie). Because PDMS is an oxygen-permeable material allowing easy fabrication, we expect to achieve specialized usability of the cubic artificial eggshell in biomedical research using engineering methods. This functionality would be difficult to realize using a tough natural opaque eggshell lacking transparency.

The development of blood vessels on the CAM could be regulated using the cubic eggshell. As demonstrated in our previous study, the area of blood vessel formation was controlled by using patterned membranes with oxygen permeable and non-permeable areas of different widths [32]. Indeed, when the width of the oxygen permeable channel is larger than 5 mm, blood vessel formation was selectively induced in the oxygen-permeable area with a bell-shaped marginal part. However, when the oxygen permeable channel width is reduced to less than 5 mm to create a smaller blood vessel formation area, we found that blood vessels could not sense the differences in oxygen permeation on the patterned surface, and blood vessels spread normally on the patterned surface. The CAM on the patterned surface was rich in blood vessels with a diameter ranging from tens of micrometers to hundreds of micrometers, which develop rapidly and become dense due to vessel branching. On the other hand, we noticed that to study vascular functions at the micrometer scale, micro-devices with a single straight channel might be sufficient instead of a branched network with dense blood vessel network [33, 34]. Therefore, it will be valuable if there is an ‘engineered’ approach to separate straight blood vessels from the complicated blood vessel networks.

In this study, by introducing a new design of the artificial cubic eggshell, we created a new platform with functionalized membranes. The growth area and direction of blood vessels on the CAM was finely controlled by constructing a lateral-side surface capable of accommodating growing blood vessels (Fig 1b). A time-lapse movie of the re-arrangement of blood vessels on CAM by the structure of the lateral-side surface is shown in S2 Movie.

## Results

### Blood vessel induction by a patterned surface on an artificial eggshell

**Fig 2:**
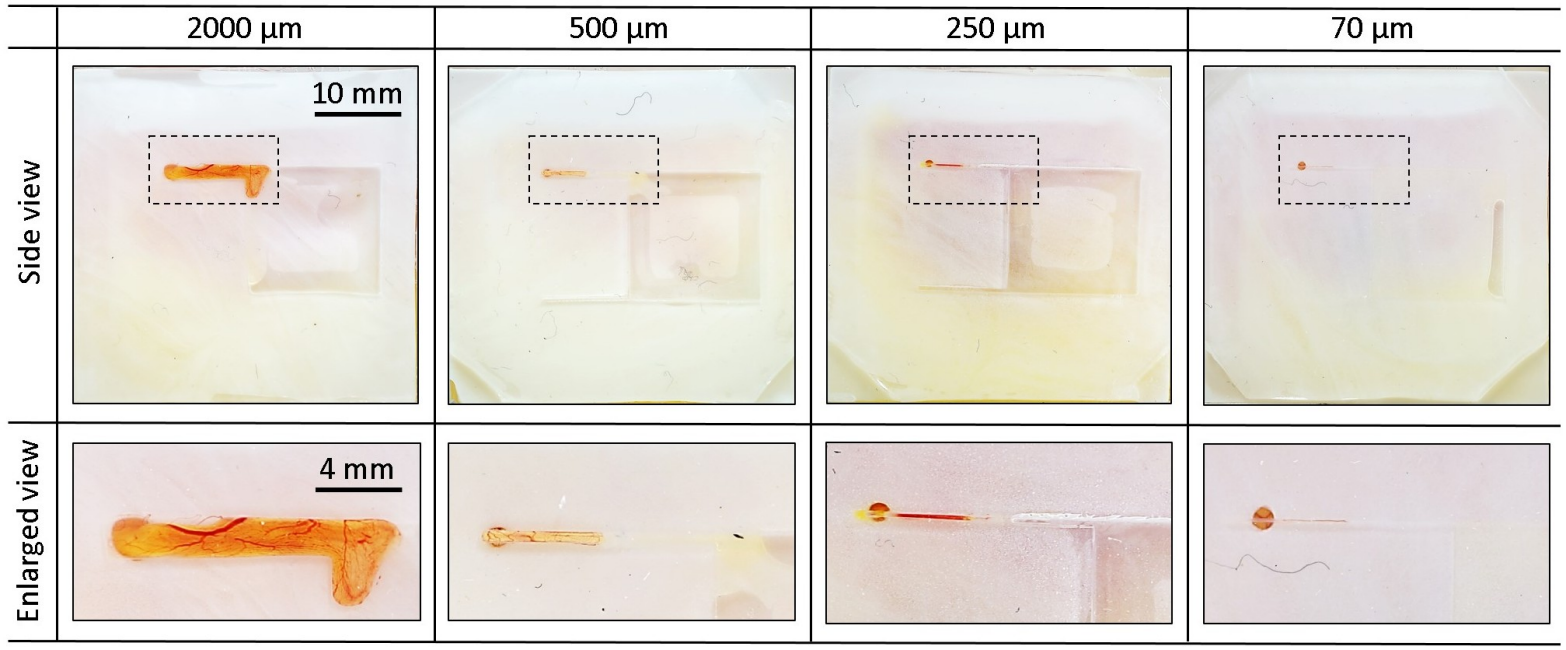
Penetration of CAM blood vessels into the interlinked inducing channels of different sizes. In order to clearly confirm the boundary of blood vessel, small amount of white ink (5 wt%) was added to a PDMS membrane. Blood vessels on the CAM were successfully induced in a horizontal direction along the orientation of inducing channels with widths of 70, 250, 500, and 2000 μm. In addition, blood vessels became straight in the inducing channels which were smaller than 500 μm.

Simply put, the patterned surface contained a small hole, an inducing channel, and an interlinked chamber. In order to confirm the effectiveness of the patterned surface, tests were conducted as shown in Fig 2. Using the patterned surface, we were able to successfully induce blood vessel formation in the channels of the culture chamber. Normally, blood vessels grow vertically from the top of a cubic eggshell to the bottom. Using the patterned surface, we were able to induce horizontal blood vessels in channels with widths of 70, 250, 500, and 2000 μm, and blood vessels in the inducing channels became straight when the width of the channel was equal to or less than 500 μm. In addition, as shown in S3 Movie, we could observe the flow of blood cells, from the channel to the inner space of the cubic eggshell. These figures show that an enclosed culture chamber with a small channel is effective for induction of blood vessels.

### Survival rate of chick embryos in cubic eggshells with a patterned surface

**Fig 3:**
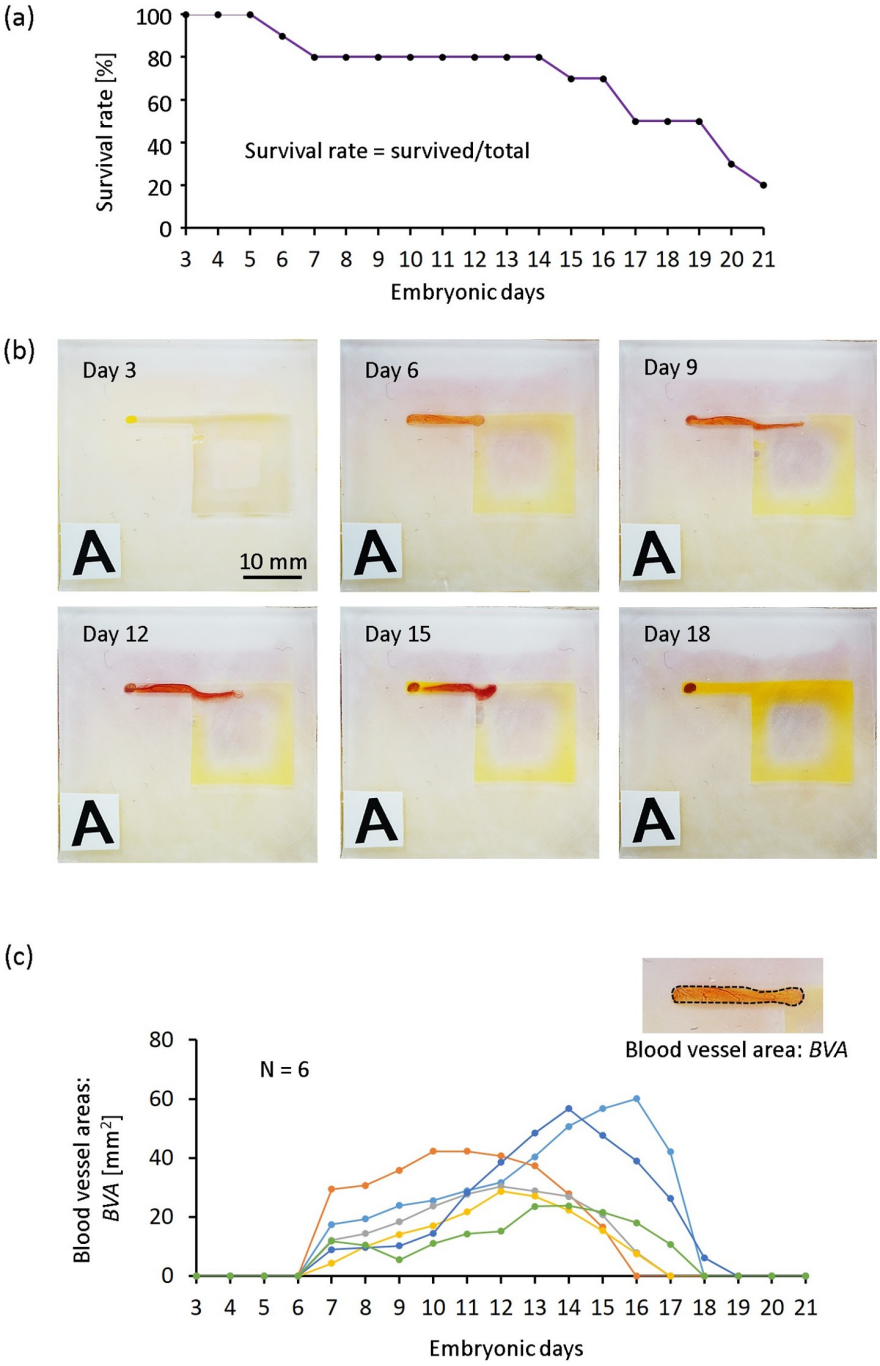
Survival rate of embryos and the penetration process of CAM with blood vessels. (a) The survival rate of embryos was investigated using cubic eggshells with a patterned surface containing a 15×15 mm^2^ air chamber with a 2-mm inducing channel. The survival rate was about 70% until day 18. (b) Typical time lapse images of blood vessel development. On day 3, induced blood vessels were not observed, and on day 7, accompanied with the rapid development of the extraembryonic circulatory system, blood vessels started to penetrate into the inducing channel. The increasing tendency of penetration was observed until about day 15. (c) Areas of blood vessel formation over time for the same cubic eggshell from day 3 to day 21 (N = 6 samples).

Since blood vessels were selectively induced into the channels of the culture chamber, a condition different from the normal physiological environment, we were concerned that the survival rate might sharply decrease after transplantation of the chick embryos. However, as shown in Fig 3a, most of the embryos remained alive for more than 15 days, and we found that the survival rate of chick embryos was appropriately 80% until day 14 and 50% until day 18. In addition, as shown in Fig 3b, the area of blood vessels increased markedly from day 7, and extended for a timespan of about 10 days (Fig 3c). Using the cubic eggshell in the current experiments, no embryo hatching was observed. From the previous studies, newly-formed blood vessels in the gelatin matrix were observed after 4 days of matrix implantation [26]. Also, we noticed that in the study by Borges et al., they established an in vivo model to study the effect of angiogenesis in tissue-engineering constructs using CAM, and incubated the cell-matrix construct for a maximum period of 10 days [28]. Therefore, our model with a patterned surface has the potential to be developed as a platform for research on angiogenesis and tissue engineering.

### Confirmation of the mechanism for blood vessel induction

**Fig 4:**
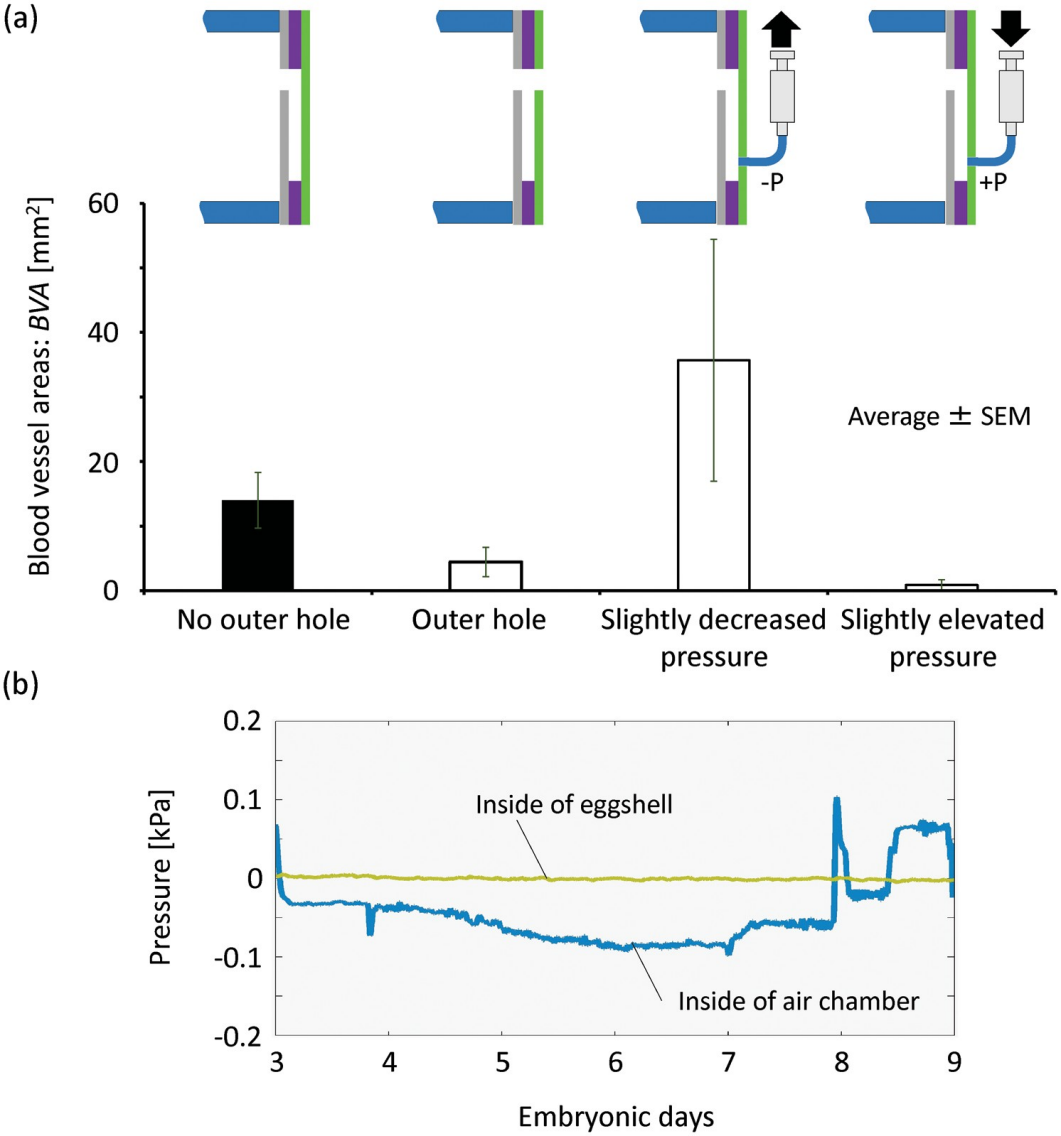
The behavior of CAM with blood vessels and pressure change in a chamber. (a) Induction experiments using an enclosed chamber, a chamber with an outer hole, a chamber exposed to negative and positive pressure were carried out to confirm the necessity for the chamber and the mechanism responsible for inducing. Blood vessel induction into a chamber with an outer hole or a chamber exposed to positive pressure was almost not observed. If controlled properly, negative pressure resulted increased CAM induction. (b) To confirm pressure change in a chamber without a hole on the outer membrane during the incubation, the inner pressure of the air chamber and the eggshell (incubator) was measured by pressure sensors (pressure gauges).

Since we hypothesized that air pressure different from the ambient environmental pressure is related to blood vessel induction, the induction experiments were carried out under four pressure conditions: 1) a patterned surface with an enclosed chamber, 2) atmospheric pressure by opening a hole on the outer membrane, 3) negative pressure, and 4) positive pressure, as shown in S3 Fig and Fig 4. A small tube having inner diameter and outer diameter 1.2 mm and 2.0 mm respectively was connected to the chamber within a patterned surface and the joint was sealed with PDMS. The other end of the small tube was connected to a syringe, which was attached with a syringe pump (YMC Co., Ltd, Japan). Negative or positive pressure inside the chamber was produced by the movement of the syringe pump at a speed of 1.0 μl/min. We calculated the blood vessel area in the inducing channels of culture chambers. With the outer hole, the induced blood vessel area decreased prominently compared to that in the chamber without an outer hole. When a small amount of air was gently sucked out of the culture chamber to produce negative pressure, a large area of induced CAM with blood vessels was observed, even larger than that in the chamber within a patterned surface normally cultured in the incubator (Fig 4). Using the pump, we have tried to suck out various volumes of air, and found that controlling of the air pressure within the chamber was extremely sensitive. Decreased pressure produced by the syringe pump always lead to collapse of the CAM and blood vessels, and the egg yolk/albumin was sucked into the chamber, as shown in S3 Fig. Dramatically elevated pressure is not suitable for blood vessel induction. However, when air inside the chamber was compressed, even when the positive pressure was just slightly higher than the atmospheric pressure, almost no induced CAM with blood vessels was observed within the PDMS channels (Fig 4). To further study the mechanism, the atmospheric pressure and the air pressure within the chamber and the pressure within the cubic artificial eggshell were measured and compared using a system with fine air pressure sensors (experiments without holes on the outer membrane). Small tubes with tips made from injection needles were used to connect the cubic eggshell to the air pressure sensors. As shown in Fig 4b, the air pressure within the interlinked chamber was not constant during the incubation period of the chick embryo. The pressure changed gradually, and an air pressure lower than the atmospheric pressure was observed at 3 days after egg insertion (day 6 or 7 after egg incubation), the time point when blood vessels spread fast on the lateral-side membrane of the cubic eggshell and begun to extend into the channels. The lower pressure within the enclosed chamber was sometimes sustained for several days. These results suggest that negative air pressure as shown in Fig 4b in the culture chamber resulting from the development process of the chicken embryo is crucial for blood vessel induction.

### Influence of the chamber size on the induction of blood vessels and design theory

We hypothesized that the volume of the culture chamber is proportional to the blood vessels induced. In order to confirm this hypothesis, we tested the effects of the chamber volume on blood vessel formation.

**Fig 5:**
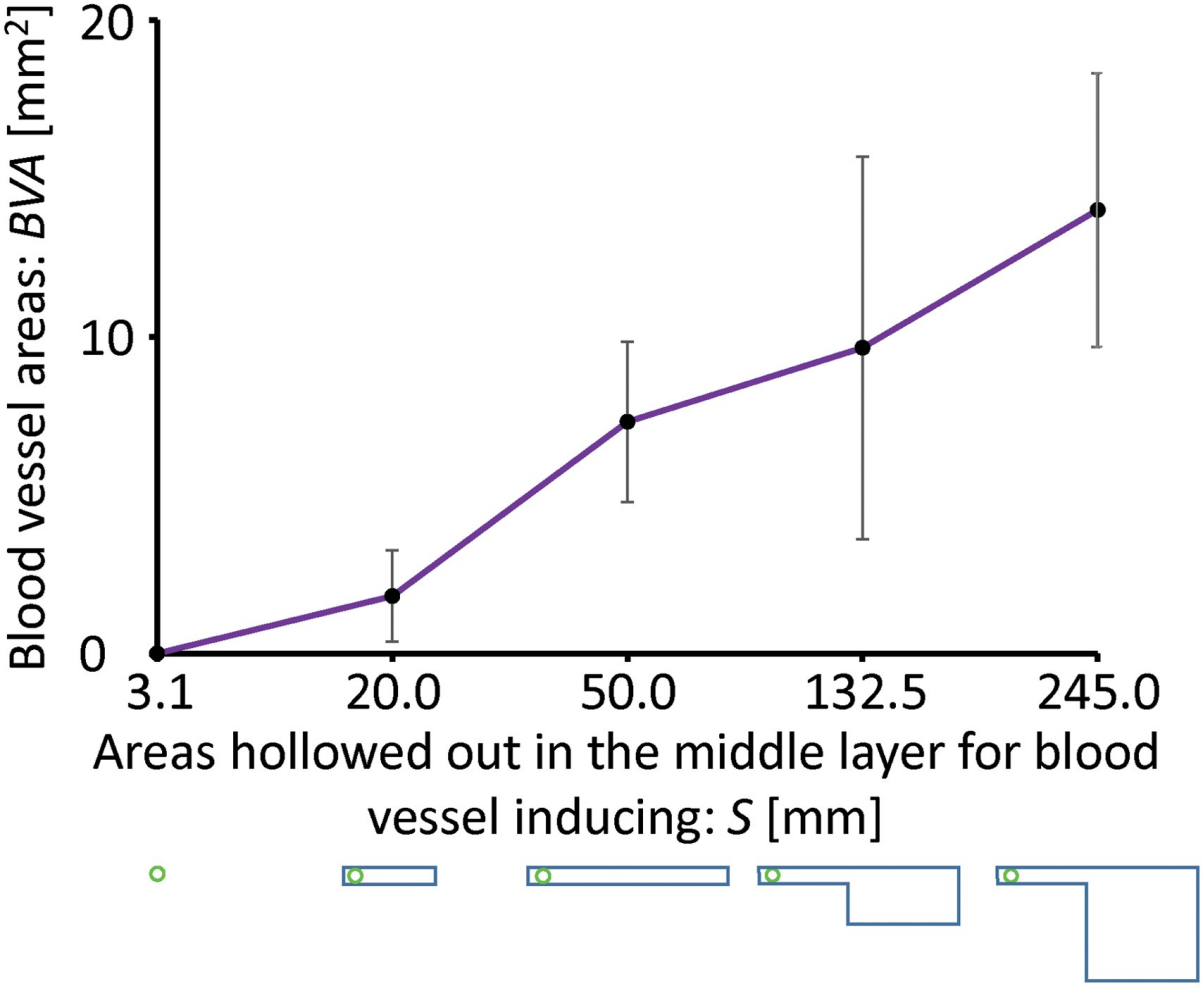
Effects of the planar occupation areas of the air chamber on the induction. The areas of blood vessel induction as a function of the planar occupation area. There is a proportional relationship between the area of induced blood vessels and the area of the air chamber. Schematic diagrams at the bottom of the graph demonstrate the planar occupation area.

First, we assessed the effects of lateral-side surfaces with middle layers of the same thickness but with different hollowed out areas to achieve different culture chamber volumes. As shown in Fig 5, the thickness of the inner, middle, and outer layer was 0.3, 0.5, and 0.3 mm, respectively. Three types of middle layers were tested, as shown in S1 Fig: (1) no culture chamber and inducing channel, (2) 2-mm-wide inducing channel only, (3) 2-mm-wide inducing channel and a 2 × 15 mm culture chamber. An inducing channel and an interlinked chamber were created by an open area in the middle layer. From these figures, we found that blood vessels did not respond to the pattern with only a small hole in the inner and middle layer. With only an inducing channel, little blood vessel area was observed. On the other hand, blood vessels were induced into the channels of culture chambers with areas of 2 × 15 mm and 7.5 × 15 mm. As shown in Fig 5, we calculated the blood vessel area in the inducing channels of culture chambers of different sizes. From the results, we found that as the area of the interlinked chamber increased, the blood vessel areas became larger, consistent with our hypothesis.

**Fig 6:**
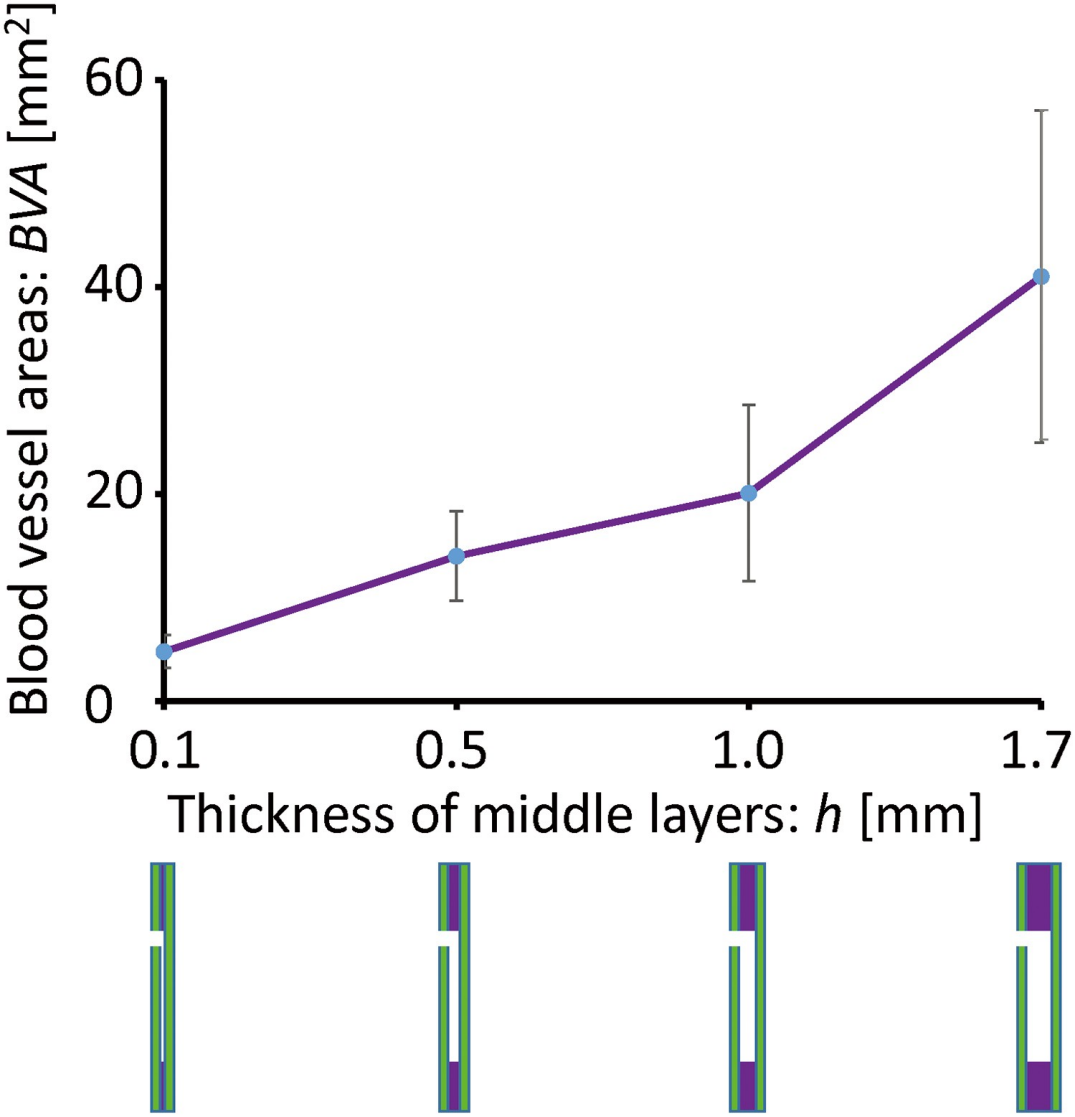
Effects of the thickness of the air chamber. The area of blood vessel formation plotted as a function of chamber thickness. An obvious proportional relationship was observed. At the bottom of the graph, a purple area sandwiched between two green areas represents the middle layer of each patterned surface.

Second, we explored the effects of lateral-side surfaces with a fixed occupation chamber area but a middle layer of different thickness. In this experiment, the areas of the culture chambers were fixed at 15 × 15 mm, and the thickness of both the inner and outer layers was 0.3 mm. The thickness of the middle layer varied from 0.1 to 1.7 mm, as shown in S2 Fig. Here, blood vessels were induced into the channels under all conditions with different thickness, and blood vessels induced into the channel of the patterned surface with a middle layer of 1.7 mm increased prominently compared to those of the pattern surfaces with thinner middle layers. From the graphed results, we found that blood vessel area in the inducing channel increased as the thickness of the middle layer increased, as shown in Fig 6.

**Fig 7:**
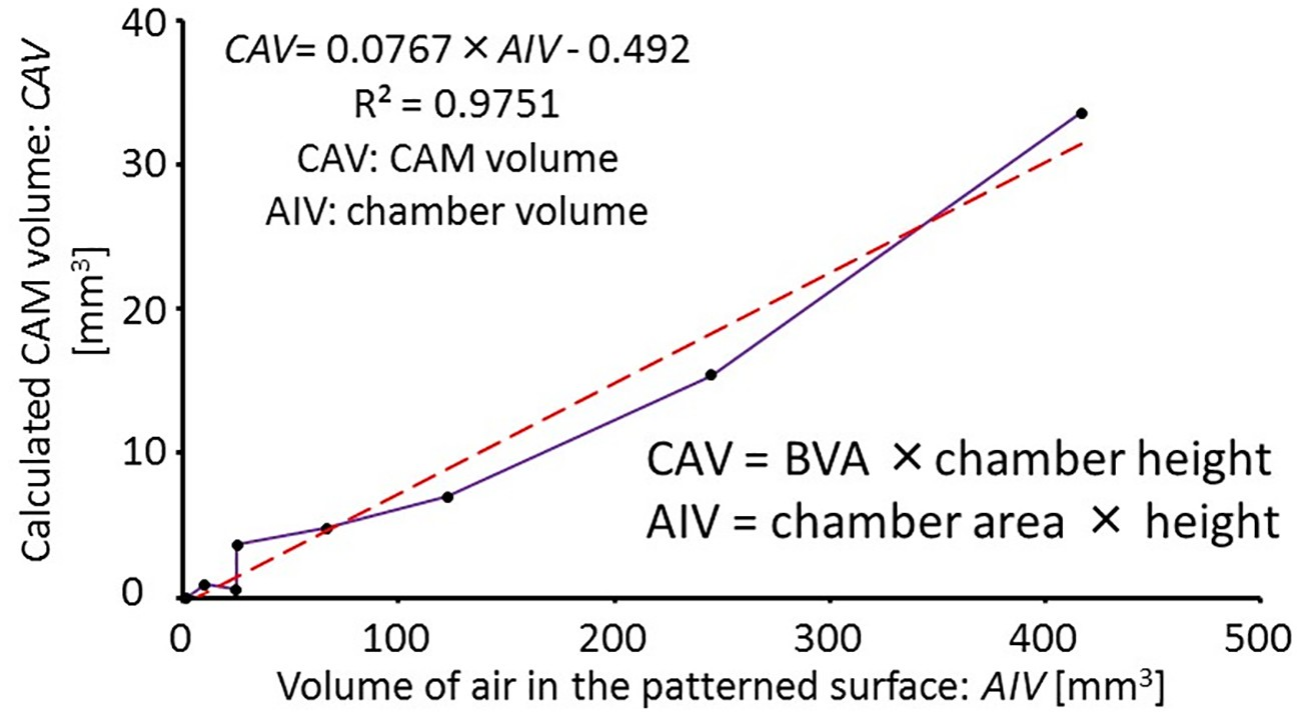
The relationship between the chamber volume and the induction of CAM blood vessels. The calculated volume of CAM blood vessels was plotted as a function of chamber volume. Similar to the results of the air chambers with different planar areas and thickness, a proportional relationship was obtained. The result suggests that a larger inducing area or inducing of blood vessels into several chambers at the same time can be realized on a lateral side membrane by increasing the thickness of the middle layer.

In order to determine the relationship between chamber size and blood vessel induction, the product of the area of the chamber and its thickness (including the channel part) was assigned to be the chamber (air) volume (AIV), and the product of averaged blood vessel area (BVA) in the chamber (including the channel part) and its thickness was regarded as the CAM volume (CAV) induced from the cubic eggshell. After calculating AIV and CAV, the relationship was obtained (Fig 7). Using linear curve fitting, we showed that the induced CAM volume was almost proportional to the volume of the culture chamber. Therefore, using the patterned method, we can design the size of the culture chamber based on the linear relationship to obtain the desired blood vessel volume.

### Components of tissues within PDMS channels

Since it is valuable to know how the blood vessels invaded into the chamber from a biological point of view, tissues within the channels were stained with H&E to observe the details of induced blood vessels. Samples were embedded in paraffin and sectioned in the direction parallel or perpendicular to the axis of PDMS channels.

**Fig 8:**
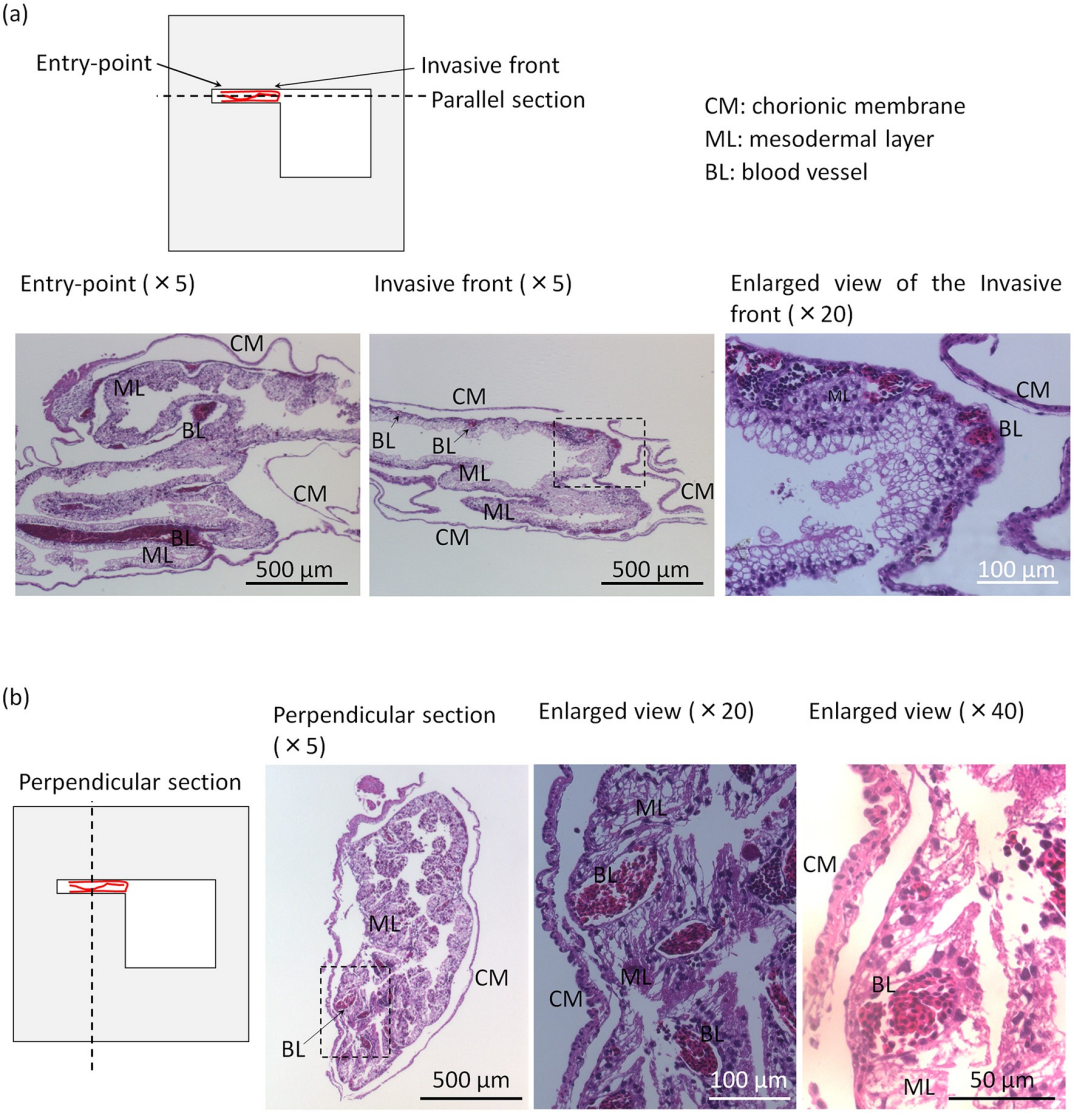
Components of CAM tissues within the PDMS channels. (a) An H&E stained longitudinal section of the induced tissue at the entry-point and the invasive front. Blood vessels were induced into the channel as a component of the mesodermal layer of chorionic membrane. Epithelial cells within the chorionic membrane were observed. (b) An H&E stained transverse section of the induced tissue. Tissue with a small hollowness surrounded by chorionic membrane and its mesodermal layer was observed. No allantoic layer was observed. Images of entry-point, invasive front, and perpendicular section: ×5 magnification; Enlarged view of (a): ×20 magnification; Enlarged view of (b): ×20 and ×40 magnification, respectively.

As shown in Fig 8a, images of longitudinal section show that the tissue, from the entry-point of the channel to the invasive front, was surrounded by a chorionic epithelial layer containing epithelial cells. Normally, the chorionic layer is in direct contact with the inner shell membrane [35]. A thick mesodermal layer containing blood vessels (the chicken’s red blood cell has a nucleus) was observed lying next to the thin stratum. Because blood vessels on CAM grew selectively on the oxygen permeable area for oxygen transportation [32], and the invasive front was exposed to air in chambers, we expected to observe enhanced blood vessel distribution within the invasive front, which may strongly pull the tissue into the channel. However, from the enlarged view of the invasive front, there was no difference, and all blood vessels were observed to be embedded in the mesodermal layer and enclosed by the chorionic membrane. Surprisingly, the allantoic layer was not observed.

Typical images of the transverse section (perpendicular to the invasive direction) are shown in Fig 8b. Consistent with the result of longitudinal section, chorionic layer and thick mesodermal layer containing blood vessels were observed. Again, there was no allantoic layer. The thickness of CAM is up to 200 μm, the mesodermal layer confined within the chorionic layer was close to each other, and the hollowness of the tissue became very small.

The results of staining further suggest that components of CAM including chorionic membrane and blood vessels responsible for gas exchange and calcium absorption [35] were induced successfully and involved in the penetration process. Allantoic membrane was not observed, and profuse growth of blood vessels was not observed. Blood vessels were just induced as a component of the mesodermal layer of chorionic membrane from the entry-point to the invasive front. Although not all the 3 layers of CAM were induced into the channels, we can achieve our goal of establishing an angiogenesis platform with patterned blood vessels and blood flows (S3 Movie).

## Discussion

There are three main advantages of the novel methodology to induce microvascular networks with blood flow into a defined and enclosed area on a patterned surface. 1) Tiny blood vessels on the CAM were successfully directed based on a new concept, although the biology of neovascularization is to be determined, and we notice that conventional methods direct new blood vessel formation using endothelial cells based on cell polarization and directional sprouting (directional migration), a complex process that remains partially understood [36–38], and induction of neovascularization in scaffolds is still challenging in tissue engineering [39]. Here, the tiny blood vessels were directionally induced into the channel from the CAM readily, and the induced blood vessels became straight when the inducing channel was smaller than 500 μm. The platform with straight blood vessels can be used a simple model to the study of vascular functions or blood flow, like the effective micro-devices with a single straight blood vessel model used in the previous studies [33, 34]. In addition, when researchers aim to establish a platform for miniaturized manipulation and fine analysis of biological cells or try to have local stimulation with physical factors on the patterned PDMS membrane using embedded microfluidic devices including parts for the application of mechanical force or electric fields [40, 41], the straight blood vessels with a defined position and direction with respect to the stimulation will simplify the system when we consider the size, direction, and functional point of the physical quantity. Although the biology of neovascularization is to be determined in the current study, our platform affords a top-down approach for guiding new blood vessel formation. 2) Low cost and simple manufacturing technique: because the inducing channels, at the micrometer scale or the millimeter scale, can be designed and fabricated easily using microfabrication techniques or conventional machining methods, the fabricated culture chamber on a lateral-side surface is a user-defined experimental platform for biomedical research. In addition, different from the 3D cell culture system with endothelialized microvessels in vitro established by Morgan et al. [42], for our system, it is not necessary to apply vascular cells such as endothelial cells or smooth muscle cells, growth factors, or extracellular matrix such as collagen or gelatin gel, to construct the platform (a culture system) with blood vessel networks. 3) Efficient use of the CAM: As the blood vessel network on the chick embryo CAM is a partial in vivo system and functions independently, there is a flow of blood cells from the heart of a live chick embryo. Therefore, instead of fabricating an in vitro circulatory system, the methodology presented in the current study applies the natural advantages of a living entity for the construction of a blood vessel-based dynamic system as the chick embryo develops. The cubic artificial eggshell highlights the usefulness of the CAM with blood flow as a promising platform for biomedical research using engineering approaches.

**Fig 9:**
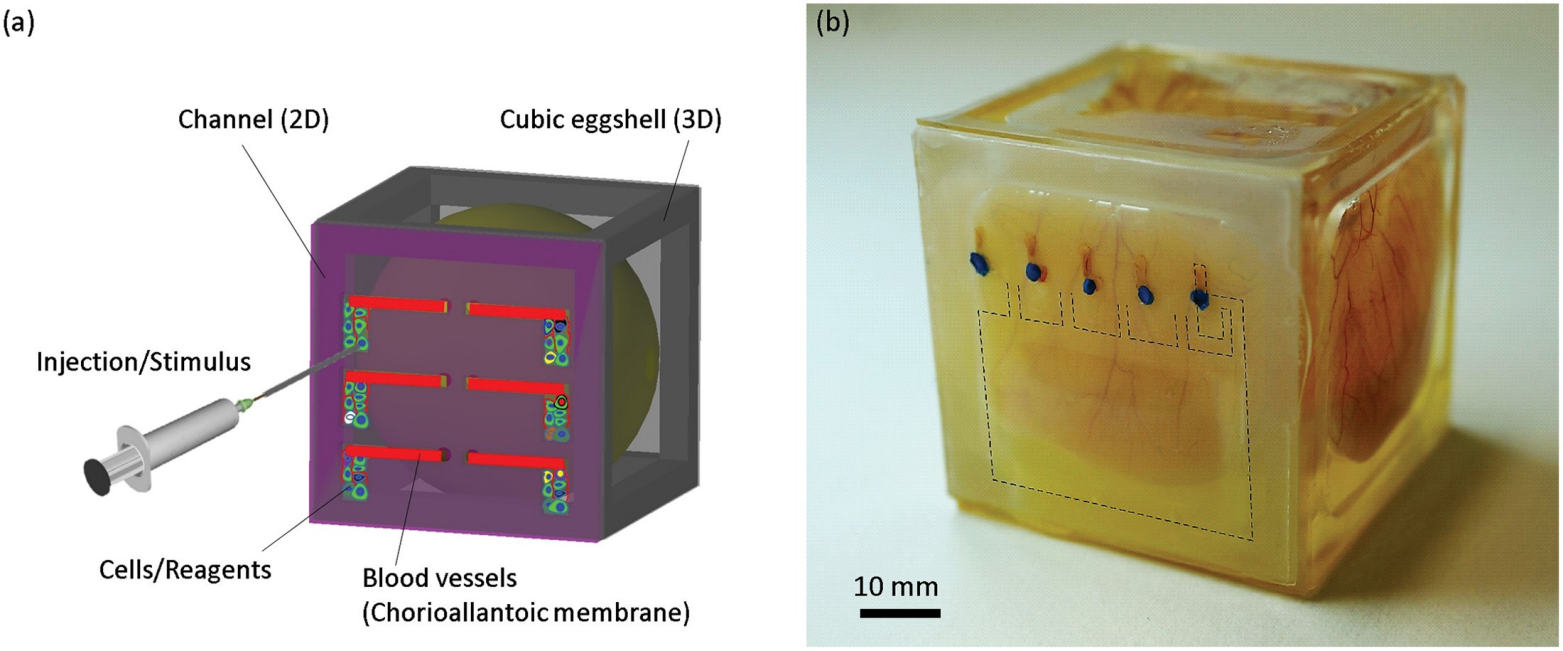
Proposed applications of the platform and an example. (a) Proposed applications of the platform containing an embryo (3D) with arrayed microchannels (2D). First, blood vessels with blood flow are induced into a few channels arranged on the lateral-side surface from the surface of an egg yolk, a nutrient source. Second, local stimulation of the circulatory system of the chick embryo can easily be achieved by channel-specific injection. (b) As an application example, we fabricated five culture chambers on a patterned lateral side surface and designed the areas of blood vessel formation for each chamber based on our design theory. Next, small holes (φ1.5 mm) were opened on the outer membrane without leakage, and water-based ink imitating drug was injected into each chamber. The release of ink within chamber was observed clearly. Because the culture chamber was an enclosed area, the injected ink was confined within each chamber without immediately influencing the other parts of the CAM. In addition, different drugs can be injected into arrayed micro-channels and comparison of drug delivery by local blood vessels is possible.

The CAM is a highly vascularized structure and has therefore been applied in various kinds of biomedical studies involving blood circulation [26, 43–49]. As mentioned above, our new design has several advantages promoting the efficient use of the CAM. Based our design theory, a patterned surface design can be used to induce blood vessels into several chambers on one lateral-side membrane as a ‘living’ high-throughput experimental platform (Fig 9a). We propose two main applications of this method in the field of biomedical research (Fig 9):

1. Local stimulation of blood vessel networks. Scientists have now established systems to do research not only on the effects of chemical factors such as growth factors but also physical factors (local physical interference), such as hemodynamic forces, on the development of blood vessels in animals [50–52]. For local stimulation by drugs or growth factors, because blood vessels on the CAM were partly induced into an enclosed chamber on the patterned surface, the blood vessels can be stimulated locally by an injection in the chamber, without dispensing drugs onto the whole area of the CAM. The system is transparent and suitable for the screening of drug transportation and the resulting response of the chick embryo. As shown in Fig 9b, to establish a platform with several channels for local stimulation, the volume of induced blood vessels was designed to be the same as that of a chamber having a planar occupation area of 15 × 15 mm and a thickness of 0.5 mm based on our design theory. Blood vessels were induced successfully, and the average volume was close to the theoretical value. Water-based ink was injected into each channel respectively, and the spreading of ink within chamber was observed clearly. As respected, the injected ink was initially confined within each chamber without immediately influencing the other parts of the CAM. Therefore, the platform can be used for drug screening, and comparison of drug delivery between channels is possible. Local forces also control the development, structure, and function of blood vessels [53]. For local stimulation by local forces, because PDMS possesses ease of fabrication and has been used extensively in a study involving microfluidics [54], a patterned surface made from PDMS with induced blood vessels is a potential platform for establishing a local stimulation system exposed to physical factors. For example, researchers could use the platform to apply mechanical stimulation to the blood vessels on CAM to control endothelial sprouting, such as the study done by Song et al. using endothelial cells within microfluidic device with micro-channels with input and output ports [55].
2. Establishment of a living system with blood flow. The method here can be used as a vascularized platform for the re-construction of tissues and organs by culturing cells on a lateral side surface of the cubic eggshell, because the results of the current study suggest that if designed properly, it is possible to induce blood vessels into multiple chambers at the same time and control the direction of the blood vessel on the patterned surface. For example, scientists have reconstructed a lung on a chip, using cells such as human airway and capillary cells and immune cells [56]. By combining microfabrication techniques and the method for directing blood vessel formation in the current study, we believe that it is possible to re-construct such a system on the CAM using several types of chick or human cells seeded into the chambers for the screening of drug toxicity such as the potency of cosmetic allergens [57] [58] [59], because the chick embryo does not naturally become immunocompetent until embryonic day 17 [16].

Oxygen consumption by the chick embryo could be a reason why the air pressure within the chamber became low, an immediate cause of blood vessel induction. The following findings support this hypothesis. 1) Based on the oxygen permeability of PDMS, oxygen diffusion in the chamber zone is locally high for blood vessel induction, because the PDMS membrane of the chamber is thinner than that of the other zone on the lateral-side surface. According to the following two studies, blood vessels on the CAM are very sensitive to differences in local oxygen diffusion. Korostyshevskaya et al. reported that after half of the eggshell is sealed, vascular reduction was observed in the chorioallantois under the sealed zone, and increased blood vessel coverage of the area under the open zone [60]. In our previous study, when we attached two polystyrene plates on the left and right sides of the lateral-side membrane to form two oxygen non-permeable areas and an oxygen-permeable middle area, blood vessels formed selectively in the oxygen permeable area [32]. 2) Oxygen permeability was necessary for blood vessel induction. When the outside of the patterned surface was attached to a plate with a low oxygen permeability to limit the local oxygen diffusion, induction of blood vessels into the channel and the chamber was not observed (data not shown). 3) A chick embryo takes in approximately 6 L oxygen for hatching during a 21-day incubation [61], and Romijn et al. reported that there was a progressive fall of partial oxygen pressure (air cell) within the egg due to the increased oxygen consumption by the chick embryo as it grew larger [62]. In the current study, low air pressure was measured only after blood vessels spread initially on the lateral-side membrane, transporting nutrients and oxygen from the lateral-side membrane [63]. 4) The area and volume of the induced blood vessels became larger as the volume of the chamber increased, containing a larger oxygen volume. Therefore, temporary low air pressure probably resulted from oxygen consumption.

The results of staining with H&E show that chronic membrane and its mesodermal layers were involved in the induced process. As we know, the CAM forms by partial fusion of chorion and allantois. The possible reasons why the allantoic layer was not observed are following. First, when CAM formed, only a part of allantois fused with chorion [64], and allantois sucked into channels was the part without fusion. Second, the fusion process was not completed when the tissue was sucked into the channel on day 6 (Fig 3b and 3c), because the allantois appeared as a balloon-like structure during embryonic day 4 or 5 [65], and fusion process occurred between day 5 and 6 of incubation [66]. However, the exact reason is still unknown, and there is a possibility that all the 3 layers of CAM can be observed within channels for a longer culture period, because what we show here are only the samples on embryonic day 8. About the role of blood vessels, an important component of CAM, because enhanced spreading of blood vessel networks was not observed within the invasive front, it is clear that the invasion (elongation) of the tissues within the channel was not led by enhanced blood vessel growth in the invasive front. Many factors may be involved in the induction process, and we know that the mechanism for inducing should be the low air pressure within the chamber (from the confirmation experiments) (Fig 4). However, the role of blood vessel sprouting in the inducing process cannot be denied, because when we make the patterned surface using an oxygen non-permeable plate with a tube for inducing, no blood vessel spreading on the surface and induced CAM was observed (data not shown). Blood vessels were not involved in invasion directly, but they involved in oxygen transportation and enhanced blood vessel sprouting on the patterned surface may lead to local negative air pressure.

## Conclusions

In the current study, we proposed a method to induce blood vessel formation in a designed chamber within a PDMS patterned surface of a cubic artificial eggshell. The results show that an enclosed chamber with negative pressure resulting from the process of embryo development is crucial for the directional induction of CAM blood vessels, and the induced CAM is proportional to the volume of the chamber. Therefore, the current method can be used in biomedical engineering research to design the area and direction of blood vessel formation according to the desired experimental conditions. Histological evaluation confirmed that CAM components including chorionic membrane and blood vessels were induced into the channels. In addition, the induction of blood vessel formation can also be realized using microfabrication techniques. Blood vessels developed in chambers having an inducing channel with a width of 70, 250, and 500 μm. Since the blood vessels with blood flow in microchannels are still alive, several culture chambers for implanted cells/tissues can be fabricated in the patterned surface during one experiment. In addition, because the cubic eggshell with patterned surfaces is transparent, chamber-specific reagent injection and screening is realized, and the development and spreading of blood vessels into the cultured tissues in the chamber can be observed clearly by eye or using a microscope. Therefore, the current method for blood vessel induction will afford a new platform for research on bioengineering, transplant biology, cancer research, and drug development.

## Materials and methods

### Ethical approval

According to the Scientists Center for Animal Welfare (SCAW) guidelines [67], studies on embryonated eggs are included in Category A of biomedical experiments, which has the lowest ethical concerns. According to the Animals (Scientific Procedures) Act 1986 [15], a chick embryo in its embryonic form is considered a protected animal from the stage of development at which half of the hatching period has elapsed. Chick embryos reach this point after a 10-day incubation, approximately halfway through the 21-day hatching period [68]. Based on these regulations, the present study was approved by the Ethics Committee of the Graduate School of Life Science and Systems Engineering (LSSE) at Kyushu Institute of Technology. Fertilized chick eggs (Sakura, Gotofuranjyo Inc.) were used in the current study. The chick embryos were incubated at 39°C with a relative humidity of 80%. Here, if it was necessary, we cultured the chick embryos in cubic artificial eggshells for up to 21 days, the time period for hatching, although hatching was not observed.

### Fabrication of the patterned surface of the cubic eggshell

The current study applied a cubic artificial eggshell as described previously [32]. Normally, the cubic eggshell was fabricated by integrating a polycarbonate (PC) frame structure and six PDMS membranes, with an unsealed upper face for the insertion of egg contents. In the current study, to obtain a fixed culture chamber for inducing blood vessels, the pattern surface was fabricated by the bonding of three PDMS membranes. The patterned surface consists of three layers: an inner layer with a small hole (in order to clearly confirm the boundary of blood vessel, small amount of white ink (5 wt%) was added to PDMS), a middle layer with a hollow part (an air chamber and a blood vessel inducing channel), and an outer layer without holes. After the three layers were fabricated, the inner, middle, and outer layers were bonded together as a lateral-side membrane that was then bonded to one face of the PC frame (S4A Fig). For the culture chamber with a channel less than 1 mm, the middle layer was fabricated using a silicon wafer with photoresist patterns. The shape and thickness of the culture chamber and the blood vessel inducing channel were designed in advance. After fabrication of the silicon wafer, liquid PDMS was poured onto the wafer slowly to fabricate the blood vessel inducing channel and the air chamber. S4B Fig shows a fabricated cubic artificial eggshell with a patterned surface.

### Preparation of eggs and insertion of egg contents

Fertilized chick eggs were purchased from a hatchery in Japan (Sakura, Gotofuranjyo Inc.). Immediately after the delivery, eggs were incubated in an incubator at 39°C with a relative humidity of 80%. According to previous studies, eggs must be transplanted to the artificial eggshell within 3 days to prevent damage induced by cracking of the eggshell and improve the embryo survival rates [22]. The contents of 3-day-cultured chick embryo were transferred to the eggshell following previously described methods [22, 69]. Briefly, first, the egg was laid horizontally for 1–2 minutes to make the embryo to lie on the top side of the yolk [22]. Second, tapped the egg gently using a sharp edge until there is a small dent on the egg [69], and carefully removed the cracked part of eggshell and the underneath inner eggshell by a pair of tweezers. Third, gently poured a small volume (approximate 5 ml) of albumin into the cubic eggshell, which provided a dampening environment to absorb shocks induced by moving the egg contents [69], pull the shells apart to open the egg, and then poured the egg contents gently into the cubic eggshell without breaking the yolk. The embryo should be on the top side of the yolk. Fourth, covered the upper face of the cubic eggshell by a PDMS membrane and kept into the incubator.

### Histological evaluation

First, an egg was cultured in a cubic eggshell with a patterned surface for 8 embryonic days. Second, the egg contents including the egg yolk, the CAM, and the embryo was removed by gently cutting off the CAM within the cubic eggshell along the patterned surface without hurting the tissue within the channel. Third, the patterned surface was removed carefully from the PC frame avoiding heavy deformation and fixed in 4% paraformaldehyde (Wako Pure Chemical Industries, Ltd., Japan) for 12 hours at 4°C. Fourth, samples within the PDMS channels were embedded in paraffin and sectioned. The samples were sectioned in both the direction parallel and perpendicular to the axis of channels. Samples were then stained with H&E and observed using inverted microscope Axiovert 200 (Carl Zeiss Corporation, Germany).

### Image acquisition, analysis, and assessment of viability

To judge the viability of chick embryos, photos and movies of chick embryos cultured in cubic eggshells were taken every 24 hours using the video mode of a digital camera (NEX-5TL, Sony Inc.).

The outline of an area of induced CAM with blood vessels was traced manually on the digital image of a patterned surface, and the area was determined using ImageJ software (NIH), a method used in many previous studies [70–72].

The time lapse S1, S2 and S3 Movies were taken with a digital microscope (Z16APO, Leica Inc.).

The chick embryo was regarded as alive based on the criteria established in the previous study [32]. Briefly, we observed the movement, beating of the heart, color of blood vessels, and the development of the extra-embryonic circulatory system.

## Supporting information

S1 Fig Design of the planar occupation areas of the air chamber on the induction. (a) to (c): induction of blood vessels on day 7 into the channels with air chambers of the same thickness but different planar occupation areas. The response of CAM with blood vessels became obvious as the planar occupation area increased.
(TIF)

S2 Fig The thickness of the air chamber for blood vessel induction. (a) to (c): the thickness of the air chambers was adjusted by increasing the thickness of the middle layer. Blood vessels developed in channels of all thickness, and the blood vessels even grew prominently into the air chamber with a thickness of 1.7 mm.
(TIF)

S3 Fig Typical images of inducing results under four different pressure conditions. The four conditions are as follows: 1) an enclosed chamber to apply pressure change induced by embryo development, 2) a hole on the outer membrane to make the pressure within chamber equal to atmospheric pressure, 3) slightly decreased pressure, and 4) slightly elevated pressure.
(TIF)

S4 Fig Schematic of the fabrication process and an eggshell with a fabricated patterned surface. (a) Three layers of PDMS membranes were bound together to form an inducing chamber on the side membrane: an inner layer with a small hole, a middle layer with a penetrating part including the blood vessel passageway and chamber, and an outer layer. (b) The contents of a fertilized egg were inserted into the cubic eggshell on embryonic day 3, and the cubic eggshell was put into the incubator for embryo culturing and blood vessel induction.
(TIF)

S1 Movie Time-lapse imaging of the growth of blood vessels on a flat lateral-side of artificial eggshell (day 3 ~ day 8). (MP4)

S2 Movie Time-lapse imaging of the growth of blood vessels on a patterned lateral-side of artificial eggshell (day 3 ~ day 8). (MP4)

S3 Movie Blood flow in the straight channel. (MP4)
