# Supplementary figures and images for: An angiogenesis platform using a cubic artificial eggshell with patterned blood vessels on chicken chorioallantoic membrane
Source: PLoS One. 2017 Apr 17;12(4):e0175595. doi: 10.1371/journal.pone.0175595 (PMC5393577; doi:10.1371/journal.pone.0175595)

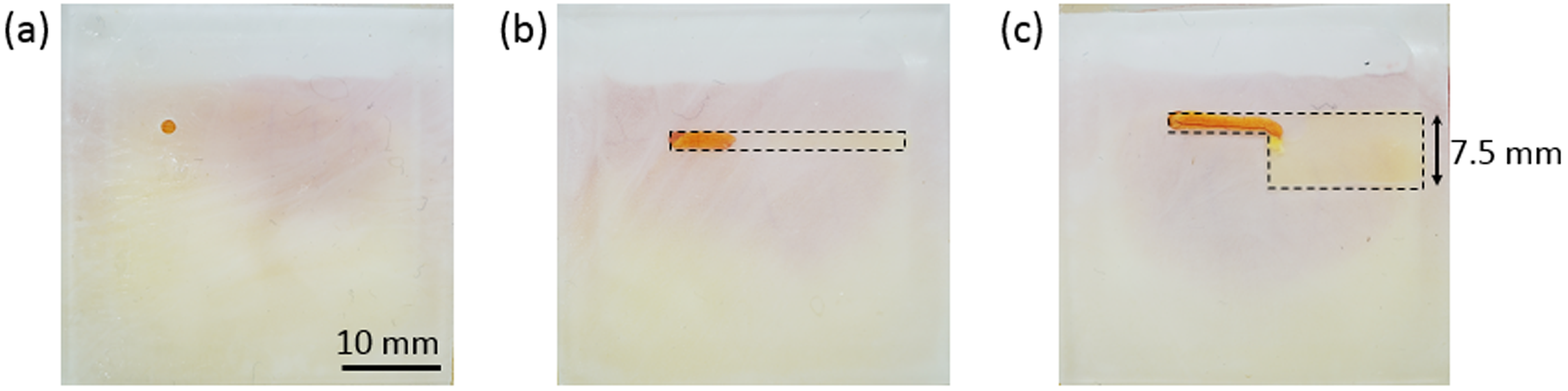

Supplement: S1 Fig — (a) to (c): induction of blood vessels on day 7 into the channels with air chambers of the same thickness but different planar occupation areas. The response of CAM with blood vessels became obvious as the planar occupation area increased. (TIF) [file pone.0175595.s001.tif]

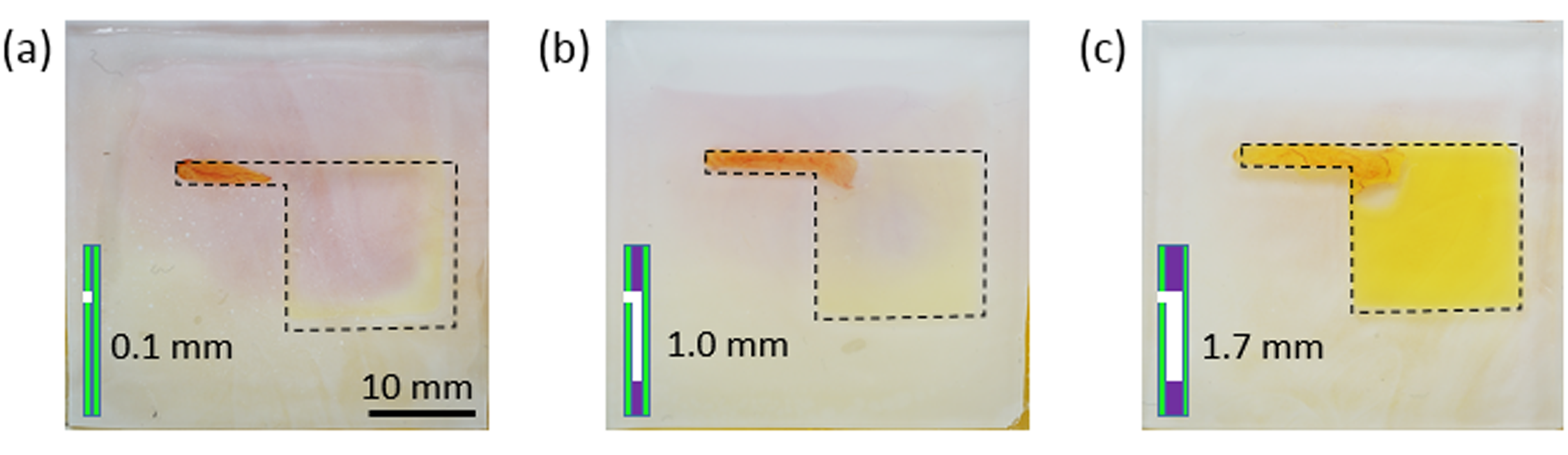

Supplement: S2 Fig — (a) to (c): the thickness of the air chambers was adjusted by increasing the thickness of the middle layer. Blood vessels developed in channels of all thickness, and the blood vessels even grew prominently into the air chamber with a thickness of 1.7 mm. (TIF) [file pone.0175595.s002.tif]

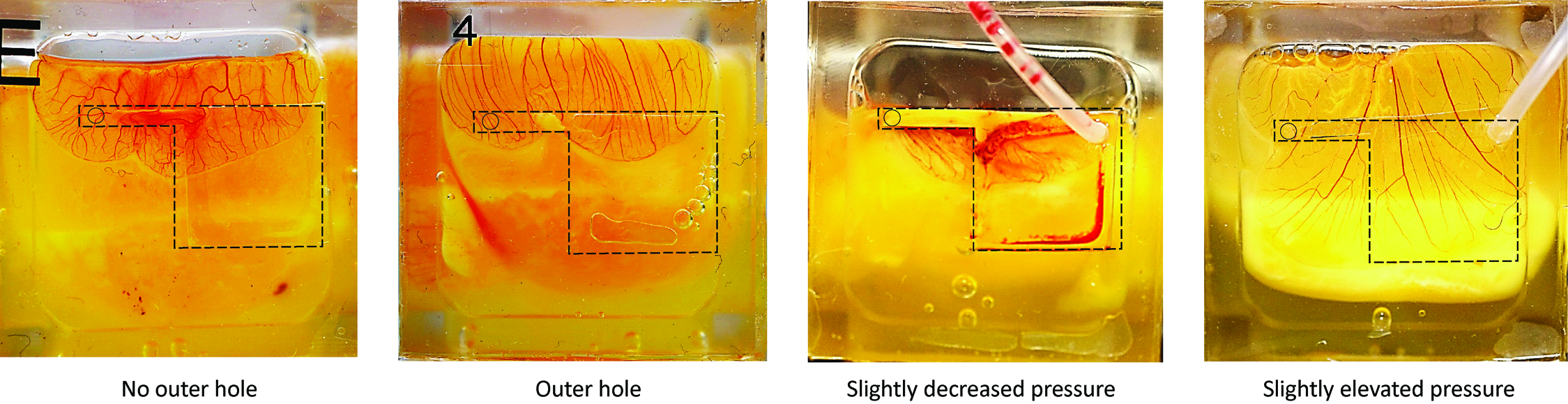

Supplement: S3 Fig — The four conditions are as follows: 1) an enclosed chamber to apply pressure change induced by embryo development, 2) a hole on the outer membrane to make the pressure within chamber equal to atmospheric pressure, 3) slightly decreased pressure, and 4) slightly elevated pressure. (TIF) [file pone.0175595.s003.tif]

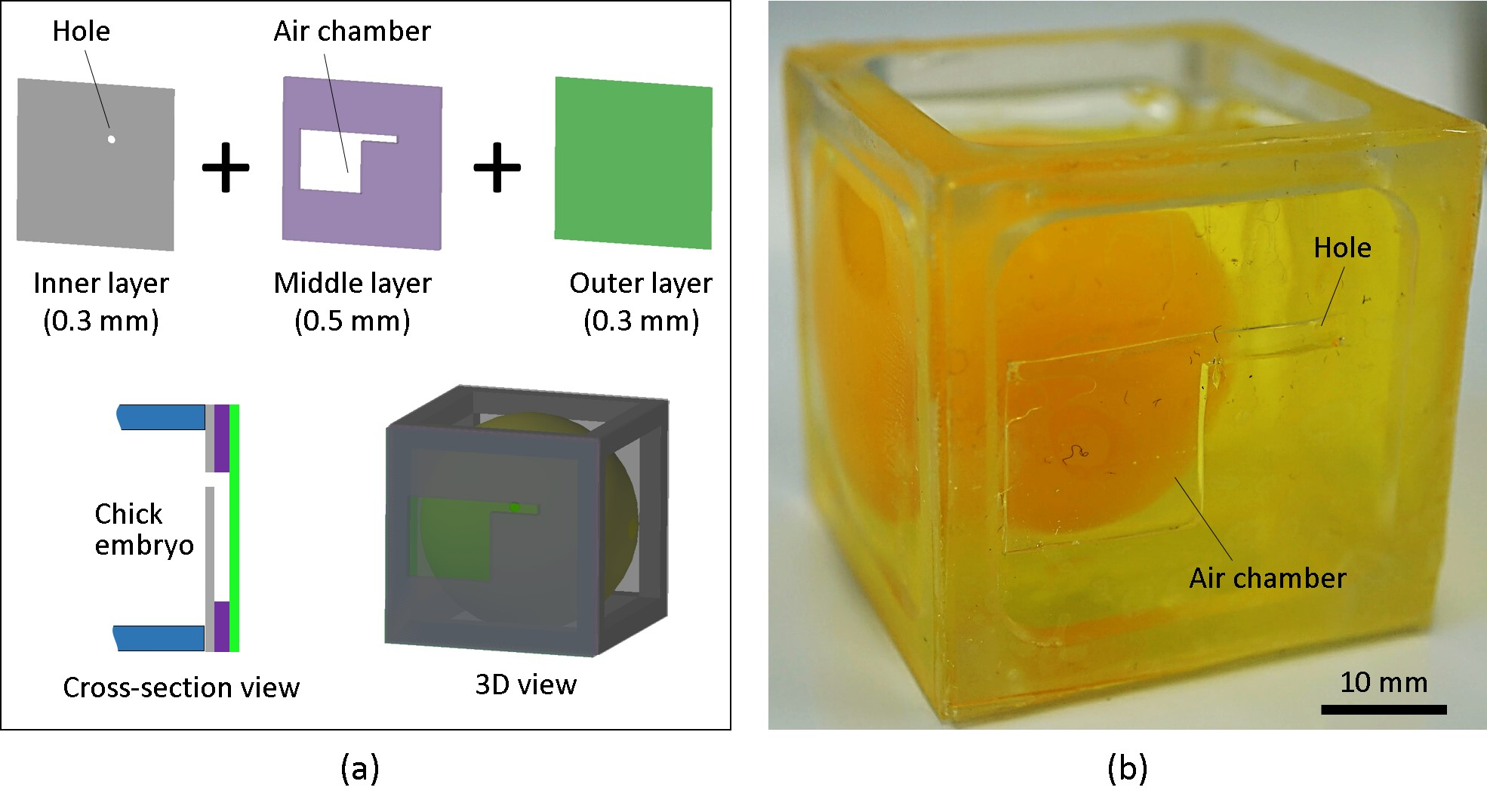

Supplement: S4 Fig — (a) Three layers of PDMS membranes were bound together to form an inducing chamber on the side membrane: an inner layer with a small hole, a middle layer with a penetrating part including the blood vessel passageway and chamber, and an outer layer. (b) The contents of a fertilized egg were inserted into the cubic eggshell on embryonic day 3, and the cubic eggshell was put into the incubator for embryo culturing and blood vessel induction. (TIF) [file pone.0175595.s004.tif]
